# Enhanced Indoor Positioning Using RSSI and Time-Distributed Auto Encoder-Gated Recurrent Unit Model

**DOI:** 10.3390/s24154815

**Published:** 2024-07-24

**Authors:** Zhe Wei, Zhanpeng Zhou, Shuyan Yu, Jialei Chen

**Affiliations:** 1 School of Computer Science, Civil Aviation Flight University of China, Guanghan 618307, China; findz@cafuc.edu.cn (Z.W.); zhouzhanpeng@cafuc.edu.cn (Z.Z.); jl_chen@foxmail.com (J.C.); 2 Yuanpei College, Shaoxing University, Shaoxing 312000, China

**Keywords:** deep learning, indoor positioning, RFID, received signal strength

## Abstract

This study presents a novel approach to indoor positioning leveraging radio frequency identification (RFID) technology based on received signal strength indication (RSSI). The proposed methodology integrates Gaussian Kalman filtering for effective signal preprocessing and a time-distributed auto encoder-gated recurrent unit (TAE-GRU) model for precise location prediction. Addressing the prevalent challenges of low accuracy and extended localization times in current systems, the proposed method significantly enhances the preprocessing of RSSI data and effectively captures the temporal relationships inherent in the data. Experimental validation demonstrates that the proposed approach achieves a 75.9% improvement in localization accuracy over simple neural network methods and markedly enhances the speed of localization, thereby proving its practical applicability in real-world indoor localization scenarios.

## 1. Introduction

Currently, the primary positioning systems are wireless positioning systems. Out-door positioning systems mainly include wireless cellular positioning systems and satellite positioning systems such as GPS and BeiDou. Indoor positioning systems use technologies like WiFi, Bluetooth Low Energy (BLE), ultrasound, and ultra-wideband (UWB). Due to the relative complexity of indoor environments, the positioning effectiveness of these technologies varies.

WiFi-based positioning systems leverage the widespread availability of WiFi infrastructure, providing high accuracy and low setup costs. BLE, known for its low energy consumption and cost-effectiveness, also offers good positioning accuracy. However, these systems face challenges in signal multipath effects and interference, which can affect their performance in complex indoor environments. UWB, with its high time resolution, offers precise positioning but requires significant infrastructure and can be costlier. According to [1], WiFi and BLE technologies have shown high accuracy in indoor environments, but their performance can be compromised by obstacles and environmental factors, which necessitates further improvement in positioning algorithms.

Accurate location awareness is crucial in various application areas and location-based services. For instance, location-based building emergency response systems can greatly enhance safety by providing the precise locations of individuals during emergencies [2]. In smart energy management, accurate positioning helps in optimizing energy usage and reducing costs [3]. Smart HVAC controls utilize location data to adjust heating, ventilation, and air conditioning systems for improved comfort and efficiency [4]. Additionally, location-based occupancy prediction enables better space utilization and energy savings by predicting and managing occupancy patterns in buildings [5]. These applications highlight the importance of improving indoor positioning technologies to meet the growing demand for accurate location awareness.

Radio frequency identification (RFID) technology, which emerged in the 1990s, is a wireless automatic identification technology. Its principle is the transmission of electromagnetic waves through magnetic field coupling or electromagnetic field coupling for information transmission, and the identification of targets through the transmitted information. Received signal strength indication (RSSI) is a commonly used method for distance estimation and positioning in wireless sensor networks due to its simplicity and cost-effectiveness [6]. 

Reference [7] proposes a hybrid RFID tag positioning method, named ReLoc, based on RSSI and phase measurements for the horizontal and vertical positioning of tags. The literature [8] develops a framework that uses RSSI and radiation patterns to determine the safe distance between drones or robots and metal objects with RFID tags. The literature [9] also proposes a robust triangulation algorithm for indoor positioning systems based on radio signal recognition to address environmental dependency. Some researchers have proposed using convolutional neural networks (CNN) for fingerprinting to estimate positions using access points. This method emphasizes the importance of weight adjustment algorithms in improving the accuracy of indoor fingerprint positioning using RSSI data, although it is computationally slow [10,11,12]. 

Reference [13] proposes a deep CNN-based method for passive RFID tag localization using combined RSSI and PDOA features, which improves on the accuracy of other methods but is prone to local optimum solutions. Therefore, finding an efficient RSSI-based RFID positioning algorithm to meet various indoor positioning requirements is of great significance.

This study makes several significant contributions to the field of indoor positioning. Firstly, it proposes the integration of an improved Gaussian Kalman filtering algorithm with a time-distributed auto encode and gated recurrent unit (TAE-GRU) network model for the first time. This novel combination enhances the preprocessing of collected signal data and improves target position localization. Secondly, the gated recurrent unit mechanism controls the information update method, reducing the risk of model overfitting, while the autoencoder in the time distribution technique better learns the temporal relationships of RSSI data for target position localization. Thirdly, experimental tests demonstrate that the proposed method improves the positioning accuracy by 75.9% compared to simple neural network methods and also significantly enhances the positioning time performance. These contributions establish the proposed method as more accurate and faster, and thus suitable for application in various positioning scenarios.

The remainder of this research is structured as follows: Section 2 delves into the related work. In Section 3, the proposed indoor localization algorithm based on the TAE-GRU model is elaborated upon. Section 4 provides a detailed description of the experiment and result analysis. Section 5 outlines future directions, while Section 6 concludes this work.

## 2. Related Work

### 2.1. Data Acquisition Equipment

An RFID system mainly comprises three core components: the reader (also known as the transceiver or interrogator), the electronic tag (or transponder), and the terminal processing unit (application software). The functioning mode of the RFID system, including the automatic identification range, is influenced by the type of reader and the type of RFID tag. These factors are interrelated and, together, form the core operational mechanism of the RFID positioning system.

#### 2.1.1. Reader Device

The core implementation module of RFID technology is the RFID reader, which can transmit in multiple frequency bands during operation, including low frequency (30–300 kHz), medium–high frequency (3–30 MHz), ultra-high frequency (300 MHz–3 GHz), and microwave frequency (greater than 3 GHz) bands.

However, the advantages and disadvantages of RFID technology do not solely depend on the frequency. Different frequencies provide different utilization methods and application extensions for the technology. Although the microwave frequency band can cover a larger identification area, its use often relies on the cooperative operation of active or semi-passive microwave RFID tags. In contrast, passive RFID tags do not require an additional power supply, so they have a longer service life, higher durability, and a relatively lower cost.

Considering these factors, we chose to use a radio frequency system with a working frequency range that is ultra-high, 860 MHz–960 MHz, and we prefer to use passive tags as the type of identification tags, with a reading frequency of 200 ms/time. This choice aims to ensure the efficient, stable, and low-cost operation of the system to meet the needs of different application scenarios.

**Figure 1:**
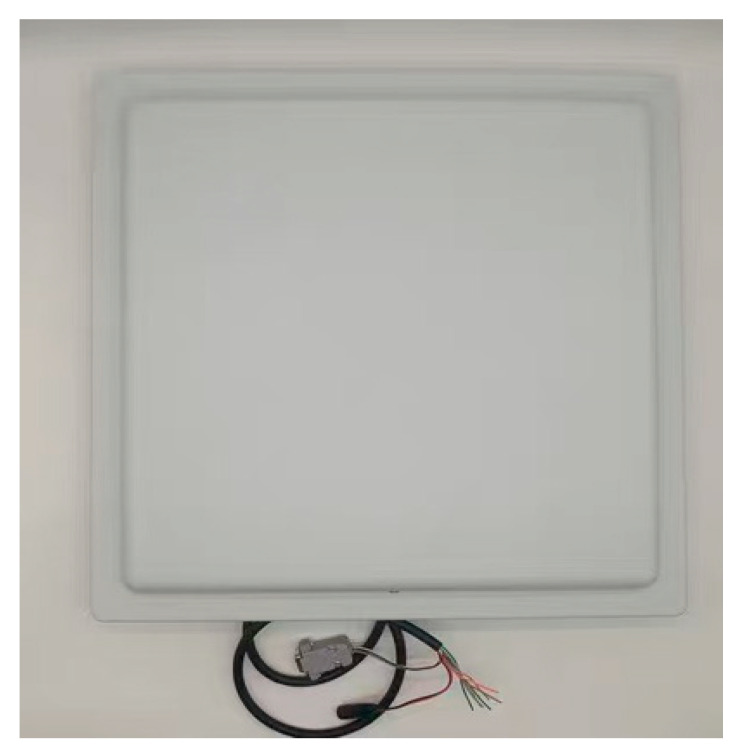
RFID reader.

The experimental reader device, provided by Shenzhen Hongguang Internet of Things Technology Co., Ltd. (Shenzhen, China), model YM190B, has the following technical features, as shown in Figure 1:

1. Antenna gain: 8 dBi circular polarization;
2. Card reading distance: 0–8 m;
3. Communication method: RS-232, Wiegand, RS485, TCP/IP;
4. Working protocol: EPC global UHF Class Gen2;
5. Working frequency: 902~928 MHz;
6. Frequency hopping method: spread spectrum frequency hopping or fixed frequency transmission;
7. Output power: 0~30 dBm;
8. Working voltage: DC 12~24 V;

#### 2.1.2. Electronic Tag

**Figure 2:**
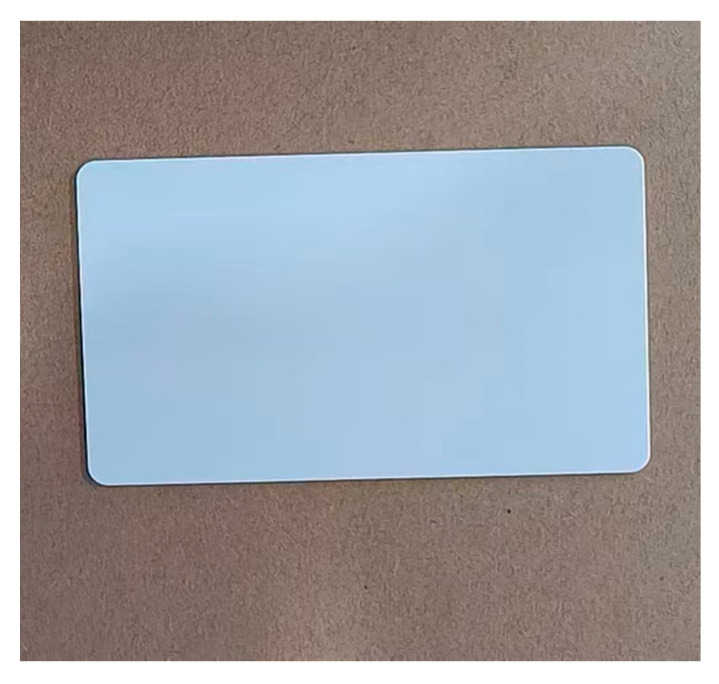
Electronic tag.

In this experiment, we used the HG-BA8554 smart tag chip from the same company. The chip meets the EPC Class1 Gen 2 RFID standard, also known as the Gen 2 standard, which is an open architecture and has received widespread support from many RFID companies. In order to meet the requirements of low processing power, low power consumption, and a low cost of the tag, improvements have been made in multiple technical aspects. These improvements include the selection of the communication frequency band bandwidth, optimization of the physical layer unit coding and signal demodulation modulation methods, the improvement of anti-collision algorithms, the device connection communication control, and the tag information protection, etc., thereby further reducing the manufacturing cost of electronic tags, as shown in Figure 2.

### 2.2. Positional Fingerprinting Method

**Figure 3:**
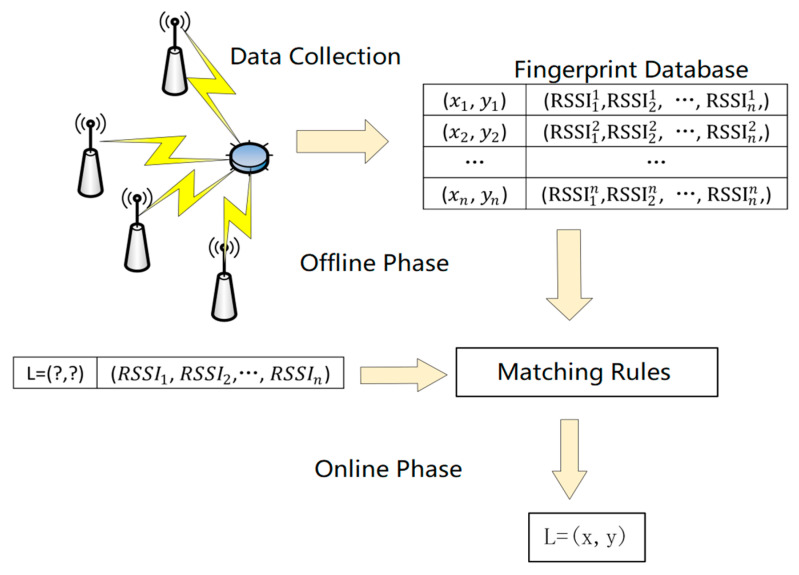
Location fingerprint method schematic diagram.

The location fingerprinting method is a positioning method based on wireless signals, as it uses the signal strength RSSI as a feature to relate to the physical location in the actual environment. By matching the RSSI information at corresponding coordinates, it determines the coordinates of unknown nodes. Figure 3 shows the working principle of the classic location fingerprinting method. 

The RSSI location fingerprinting method is a commonly used indoor positioning method, typically including two stages: offline training and online testing [14].

In the offline fingerprint stage, the indoor environment is divided into multiple reference points, which are usually evenly distributed throughout the positioning area. Then, using receiver devices to measure signal values from different wireless access points at each fixed position, the collected RSSI vectors and other feature information are combined into a fingerprint and saved in a fingerprint database, forming the offline fingerprint. To improve accuracy, the RSSI vectors are usually collected multiple times over a period and averaged to reduce the impact of environmental interference and errors on the fingerprint.

In the online positioning stage, the positioning target needs to carry an RSSI collection device and collect RSSI vectors from the surrounding environment in real-time while moving. The collected real-time RSSI vectors are matched with the data in the offline fingerprint database, typically using matching algorithms to find the reference points that best match the real-time data. The matching algorithm calculates the similarity between the real-time data and each reference point in the fingerprint database, selecting the set of reference points with the highest similarity as possible positioning results. Finally, through further calculation and processing, the position coordinates of the positioning target can be obtained.

### 2.3. Filter Preprocessing

Due to various factors such as the indoor temperature, humidity, shadowing, fading, and multipath effects, in most cases, only high-noise signal strength measurements between nodes can be used to solve node localization problems. The received signal-strength indication values between nodes fluctuate significantly over time and are easily affected by various interferences. This fluctuation and interference results in a large amount of abnormal data in the received RSSI values. This issue affects the use of wireless sensor network platforms and passive RFID technology, exacerbating inaccuracies.

To reduce the impact of these abnormal data on the localization accuracy, improve the quality of target localization information collection, and make reasonable use of the received signal strength indicator, we analyze some filtering techniques for data preprocessing. The aim is to minimize the influence of noise on the RSS location estimation during the measurement process and reduce localization errors caused by fluctuations in the signal strength in the indoor environment.

#### 2.3.1. Mean Filtering

Mean filtering model refers to the process of creating new data point sequences by the weighted averaging of multiple RSSI datasets received by nodes from another node. The datasets typically have a fixed length, and new points are added to the original data while excluding the oldest data points to create the new set. The mean filtering algorithm assigns equal weights to all data points. In RSSI filtering, when samples do not share any concept of relative importance, their weights can be considered equal and a simple moving average filter can be applied, represented as:

(1) $$\begin{array}{c} \bar{RSSI}=\frac{1}{N}\overset{N}{\underset{i=1}{\sum }}RSSI_{i},\; \end{array}$$

In this formula, $\bar{RSSI}$ represents the moving average RSSI value, which smooths out the original RSSI data. N denotes the window size, i.e., the number of data points used for averaging. $\sum_{i=1}^{N}RSSI_{i}$ indicates the sum of the first N RSSI values within the window, which are selected from the original data. 

Mean filtering is a simple and effective method, particularly suitable for scenarios with large data sample sizes and small signal fluctuation ranges. In such cases, mean filtering can effectively improve the signal smoothness and reduce fluctuations caused by interference. However, the credibility of mean filtering decreases when the RSSI signal values fluctuate strongly. This is because mean filtering simply averages the RSSI values over a period of time. If the signal fluctuates greatly, the average value may not accurately reflect the true changes in the signal, resulting in a significant deviation between the filtered result and the actual value.

#### 2.3.2. Gaussian Filtering

By establishing a Gaussian distribution model based on multiple RSSI values obtained from the same node [15], and utilizing the characteristics of the Gaussian distribution, RSSI values that meet high probability requirements are collected as valid values. The geometric mean of these values is then calculated to achieve RSSI value filtering. The probability density function for measuring signal amplitudes that follow a Gaussian distribution is:

(2) $$f\left(x\right)=\frac{1}{\sqrt{2\pi }\sigma }e^{-\frac{{\left(x-\mu \right)}^{2}}{2\sigma^{2}}},$$

among which

(3) $$\begin{array}{c} \mu =\frac{1}{k}\overset{k}{\underset{i=1}{\sum }}x_{i}, \end{array}$$

(4) $$\begin{array}{c} \sigma^{2}=\frac{1}{k-1}\overset{k}{\underset{i=1}{\sum }}{\left(x_{i}-\mu \right)}^{2}. \end{array}$$

Gaussian filtering, as a commonly used signal processing method, is particularly suitable for handling signals that are interfered with and lack stability. This algorithm calculates the weighted average of signal values, effectively reducing the impact of noise and improving the signal accuracy and stability [16].

However, Gaussian filtering also has some limitations. Firstly, the effectiveness of the algorithm depends on the number of samples in the dataset. If the number of samples is insufficient, its filtering effect will be significantly reduced, and it may not effectively remove noise [17]. Secondly, Gaussian filtering performs less effectively in handling long-term interference issues, especially shadowing effects, energy reflections, and other long-term interferences. Additionally, if the processing time for sample signals is too long, it may affect the real-time nature of positioning, making it unsuitable for scenarios requiring immediate response.

#### 2.3.3. Kalman Filtering

When RFID tag signal strength RSSI is used as the research object, ideally, the RSSI values of the tags should remain stable and unchanged, meaning the current RSSI value in the signal should be consistent with the RSSI value from the previous moment. However, in practical applications, due to various interference factors, there is often a deviation between the RSSI value and the true value, typically manifesting as Gaussian white noise [18]. Traditional filtering algorithms often face challenges in terms of their tracking capability and adaptability when dealing with dynamically biased data. Especially when the system’s motion state changes, the filtering accuracy of these algorithms often significantly decreases. They typically rely on fixed- or variable-length windows to fill in missing data, but improper window size selection may lead to issues such as positive and negative readings, resulting in unsatisfactory filtering effects [19].

According to the principles of Kalman filtering, at any given moment, two RSSI values are obtained: one is the model-based predicted value, and the other is the value obtained through actual measurement. By cleverly combining these two values and their respective noise characteristics, Kalman filtering can calculate RSSI values that are closer to the real situation. This feature makes Kalman filtering widely applicable in RFID indoor positioning systems, contributing to improved positioning accuracy and stability [20].

By measuring the actual RSSI values of the RFID system, calculating RSSI estimation values, and recursively processing according to the prediction-correction model, random noise can be eliminated, thereby reconstructing the system state [21].

Estimating the current state of RSSI:

(5) $$\begin{array}{c} \bar{RSSI}\left(\frac{k}{k-1}\right)=RSSI\left(\frac{k-1}{k-1}\right), \end{array}$$

In the above equation, $RSSI\left(\frac{k-1}{k-1}\right)$ represents the optimal prediction at time *k* − 1, and $\bar{RSSI}\left(\frac{k}{k-1}\right)$ denotes the predicted value at time *k*.

The calculation of the current-state preliminary estimate involves using the previous state value and the error covariance control matrix. In the correction phase, the current observation is combined with the estimated state value to correct and predict the latest state value. During the update step, the error covariance *P* and the Kalman gain $K_{g}$ are calculated as follows:

(6) $$\begin{array}{c} P\left(\frac{k}{k-1}\right)=P\left(\frac{k-1}{k-1}\right)+Q, \end{array}$$

(7) $$K_{g}\left(k\right)=\frac{P\left(\frac{k}{k-1}\right)}{P\left(\frac{k-1}{k-1}\right)+R},$$

In the above equations, $P\left(\frac{k}{k-1}\right)$ represents the covariance of $\bar{RSSI}\left(\frac{k}{k-1}\right)$, and $P\left(\frac{k-1}{k-1}\right)$ represents the covariance of $\bar{RSSI}\left(\frac{k-1}{k-1}\right)$. Q denotes the covariance generated by the noise present in the signal system.

Optimal estimation of the RSSI value at time *k*:

(8) $$RSSI\left(\frac{k}{k}\right)=\bar{RSSI}\left(\frac{k}{k-1}\right)+K_{g}\left(k\right)\left(RSSI\left(k\right)-\bar{RSSI}\left(\frac{k}{k-1}\right)\right),$$

Continuously repeating the above steps recursively allows us to obtain the optimal RSSI value, thus achieving signal filtering.

#### 2.3.4. GF-KF Filtering Improvement Algorithm

When measuring RSSI signal distances, considering the various factors that affect RSSI signals, single Gaussian or Kalman filtering alone cannot completely eliminate wide fluctuation points. Therefore, the GF-KF filtering improvement algorithm is proposed.

The filtering algorithm steps are as follows:

1. Collect RSSI values and represent them as $y=\left\{RSSI_{1},RSSI_{2}\cdots RSSI_{n}\right\}$;
2. Apply Gaussian fitting to filter the data, resulting in $y_{1}=\left\{\bar{RSSI_{1}},\bar{RSSI_{2}}\cdots \bar{RSSI_{n}}\right\}$;
3. Calculate the mean and standard deviation of $y_{1}$:
  (9) $$\begin{array}{c} E\left(y_{1}\right)=\frac{1}{N}\overset{N}{\underset{i=1}{\sum }}RSSI_{i}, \end{array}$$
  (10) $$\begin{array}{c} \sigma \left(y_{1}\right)=\frac{1}{\sqrt{N}}\sqrt{\overset{N}{\underset{i=1}{\sum }}{\left(\bar{RSSI_{i}}-E\left(y_{1}\right)\right)}^{2}}. \end{array}$$
4. Check if $\left|\bar{RSSI_{i}}-E\left(y_{1}\right)\right|<2\sigma \left(y_{1}\right)$. If not satisfied, set *N = N* + 1 and go back to step 2 to perform Gaussian fitting again. Proceed to step 5 when the condition is met;
5. Apply the Kalman filtering algorithm to $y_{1}$, resulting in $y_{2}$;
6. Perform linear regression on $y_{2}$, and output the result as $y_{3}$.

## 3. Indoor Localization Algorithm Based on TAE-GRU Model

Due to the fact that the positioning method in this paper falls within the realm of fingerprint positioning, it is necessary to place the input and output values on a standardized scale for ease of use in positioning. Standardization is achieved through segmentation, where the input and output vectors meeting Euclidean standard values are stored for conversion by preprocessing the input vector. The dataset is then divided into training and testing sets to implement the model’s location positioning in two stages: offline and online. During the offline stage, the RSSI readings of each reference location tag are collected into a database for better application of the neural network model in positioning. During the online stage, the input data are used for feature learning and parameter training of the model, completing the location classification and ultimately achieving location matching positioning.

### 3.1. Recurrent Neural Network

The recurrent neural network (RNN) algorithm utilizes directed cycles formed by connections between units to process sequential data in deep neural networks. Therefore, the output learned using the RNN model is characterized not only by the current input but also by all historical input data. Just as convolutional neural networks (CNNs) are specialized in processing grid-like data, RNNs focus on analyzing serialized data, such as a neural network of $x^{\left(1\right)},\cdots ,x^{\left(\tau \right)}$. RNNs are typically used in cases where there is a sequential, continuous relationship between the input and output data with respect to time [22].

Compared to traditional neural networks, recurrent neural networks (RNNs) have loop connections that allow states to be transmitted between different time steps. Each output not only depends on the current input but also on the previous states. Therefore, RNNs are particularly adept at handling sequential data tasks such as natural language processing, time series prediction, and speech recognition [23]. From a temporal processing perspective, RNNs use internal recurrent connections to capture dynamic behaviors and long-term dependencies in sequence data. Through recursive computation and internal state transmission, RNNs can remember information from previous time steps, exhibiting memory capabilities. This mechanism allows the model to share the same set of parameters across different time steps, making it more efficient, as the number of parameters remains independent of the sequence length.

**Figure 4:**
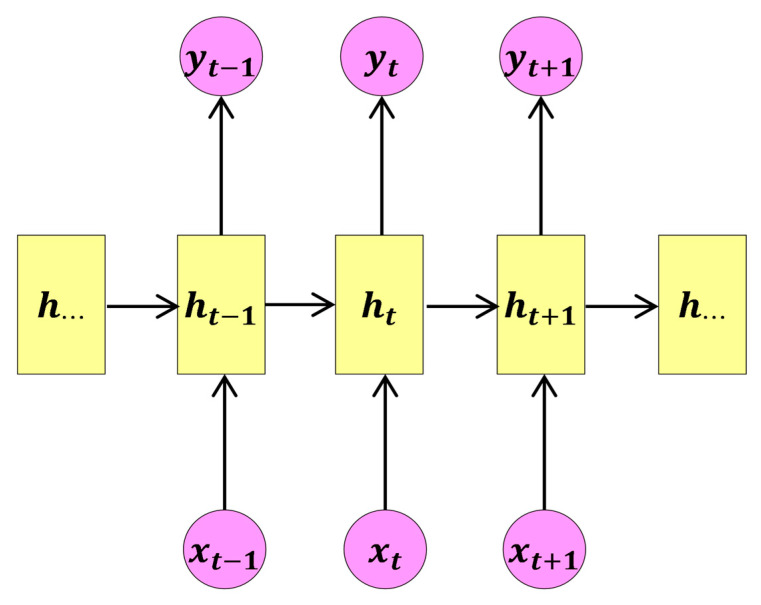
Recurrent neural network architecture diagram.

As shown in Figure 4, the basic structure of a recurrent neural network (RNN) includes an input layer, hidden layer, and output layer. Each layer of the RNN consists of multiple neurons, with information transmitted between layers via weighted connections. The hidden layer combines the current input state with the previous state to capture dynamic temporal patterns in sequential data.

In the RNN, multiple neurons between each layer transmit information via synapses and connect the layers through a weight matrix. This structure enables the RNN to effectively process sequential data and capture dynamic temporal dependencies. The input states in the temporal window of the feedforward structure of the RNN are temporally spatialized, enabling the previous state to be remembered as a recurrent unit. In the RNN, the recursive formula is applied to the time state and the previous state in the model’s memory to process the vector sequence [24].

When RNN has input $x_{t}$, it computes the current state $h_{t}$ at time t based on the activation function $f_{w}$, using the previous state $h_{t-1}$, as follows:

(11) $$h_{t}=f_{w}\left(h_{t-1},x_{t}\right)=\tanh\left(W_{hh}h_{t-1}+W_{xh}x_{t}\right),$$

(12) $$y_{t}=W_{hy}h_{t}.$$

In the traditional concept of RNNs, gradient signals are eventually multiplied by the weight matrix of neurons in the recurrent hidden layer during the backpropagation phase. The magnitudes of weights in the transition matrix can significantly impact the learning process. This matrix of weights can lead to two problems: if the weights are small, this may cause the gradient to vanish, slowing down or halting learning; another problem is gradient explosion, which occurs when the weights in the matrix are large.

Therefore, the significant gradient signals in this application can lead to learning divergence. These two problems necessitate the further improvement and design of recurrent neural networks for RSSI indoor positioning, utilizing gated recurrent unit networks to achieve accurate outputs.

### 3.2. Long Short-Term Memory Network

**Figure 5:**
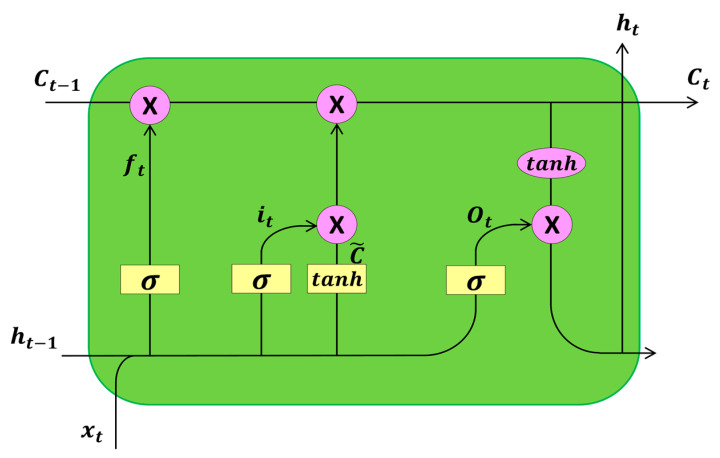
Long short-term memory network structure.

Long short-term memory (LSTM) networks introduce a new structure called a memory cell, which consists of four components: an input gate, forget gate, output gate, and self-recurrent connection neuron [25]. The input gate can alter the state or block the storage unit with the help of the input signal. Self-recurrent connections are configured to prevent any external interference. The forget gate regulates the self-recurrent connection to ensure that the unit remembers or forgets its previous state. Finally, the output gate allows changes to other neurons based on the current state of the storage unit [26]. Figure 5 depicts the long short-term memory network structure.

Each unit in the LSTM network block has an internal cell state $c_{t}$ and a hidden state $h_{t}$. The parameters are updated based on the network input $x_{t}$ at time t and the previous hidden state $h_{t-1}$ as follows:

(13) $$c_{t}=f⊙c_{t-1}+i⊙g,$$

(14) $$h_{t}=o⊙\tan h\left(c_{t}\right),$$

where ⊙ represents element-wise multiplication, o is the output gate modulating the exposure level of the storage contents, i is the input gate controlling the input volume of the unit status candidates, f is the forget gate controlling the unit status at the previous time step to forget the unit, and o is the output gate computing the hidden state of the current time step. These parameters can be computed using the following formula:

(15) $$\left(\begin{array}{c} i\\ f\\ o\\ g \end{array}\right)=\left(\begin{array}{c} \sigma \\ \sigma \\ \sigma \\ tanh \end{array}\right)W\left(\begin{array}{c} h_{t-1}\\ x_{t} \end{array}\right),$$

where W is the weighted matrix, σ is the sigmoid function, and tanh is the hyperbolic tangent function. The size of the network input $x_{t}$ corresponds to the number of input features $N_{feat}$. The sizes of the hidden state vector $h_{t}$ and the internal unit states $c_{t}$, i, f, o, and g are determined by the number of hidden units (NHU) of the unit, which controls the amount of information remembered between time steps. Finally, the size of the weighted matrix W is $\left(4\cdot \mathrm{NHU}\right)X\left(N_{feat}+\mathrm{NHU}\right)$.

LSTM units, unlike traditional recurrent units that rewrite their content at each time step, can decide whether to retain existing memory by introducing gates. Intuitively, if an LSTM unit detects an important feature early in the input sequence, it can easily carry that feature information over long distances, thus capturing potential long-range dependencies.

### 3.3. Gate Recurrent Unit Network

**Figure 6:**
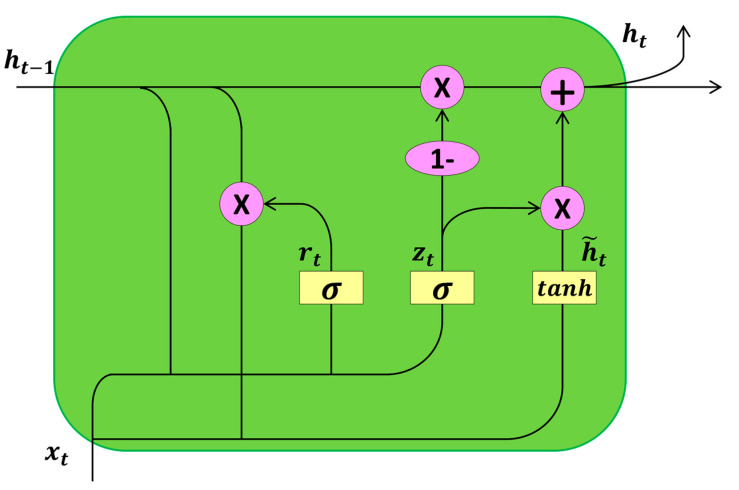
Gated recurrent unit network structure.

GRU is a gating mechanism introduced in recurrent neural networks, where each recurrent unit adaptively captures dependencies at different time scales. Similar to LSTM units but structurally simpler, the GRU comprises only an update gate, a reset gate, and a hidden state, without a separate memory unit. Therefore, considering RFID signals as a sequence data type, GRU outperforms LSTM units in terms of training convergence, parameter updates, and generalization for location estimation. Figure 6 shows the internal structure of the gated recurrent unit network.

The input to the GRU is transformed by reset gate r, which determines how much information from the previous GRU unit should be forgotten, and update gate *z*, which determines how much information should be passed from the current GRU unit to the next GRU unit. Their calculations are as follows:

(16) $$r_{i}=\sigma \left(\left[W_{r}x_{i}\right]+\left[U_{r}x_{i,t-1}\right]\right),$$

(17) $$z_{i}=\sigma \left(\left[W_{r}x_{i}\right]+\left[U_{z}h_{t-1}\right]\right),$$

where W and U represent the weights of the GRU, and *h* is the previous hidden state. The current hidden state is computed as follows:

(18) $$h_{i,t}=z_{i}h_{i,t-1}+\left(1-z_{i}\right){\tilde{h}}_{i,t-1},$$

(19) $${\tilde{h}}_{i,t-1}=tanh\left(\left[W_{h}x_{i}\right]+[U_{h}\left(r_{i}⊙h_{i,t-1}\right)]\right).$$

For the reset gate, a larger value of $r_{i}$ means that much information from the previous unit is forgotten. For the update gate, a larger value of $z_{i}$ indicates that more information is being passed to the next unit.

### 3.4. Temporal Distribution Autoencoder—Gated Recurrent Unit Model

**Figure 7:**
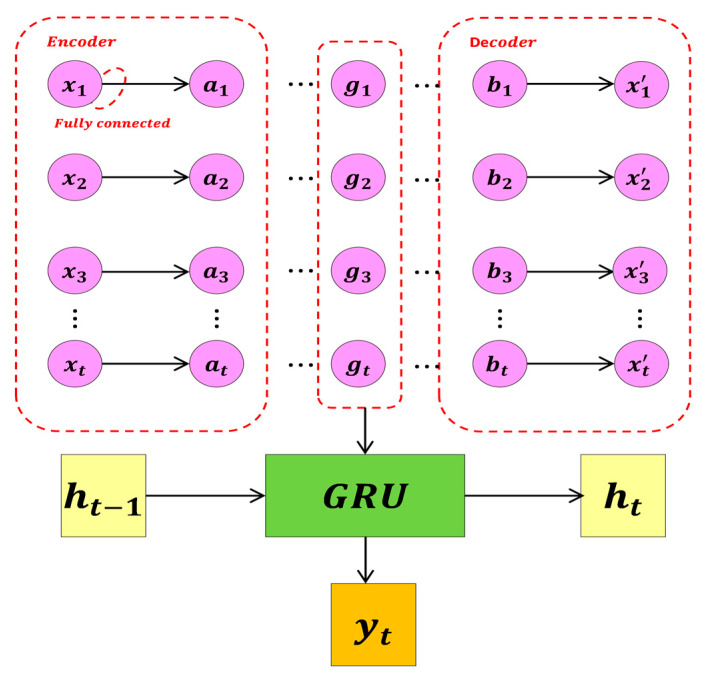
Temporal distribution autoencoder–gated recurrent unit network structure.

The temporal distribution autoencoder model technology is used to reconstruct RSSI data to improve the accuracy of location positioning. Figure 7 illustrates the structure of the model, which consists of an encoder part and a decoder part, both belonging to the temporal distribution autoencoder.

The encoding process of sample data from the input layer to the hidden layer:

(20) $$h=g_{\theta_{1}}\left(x_{i}\right)=\sigma \left(W_{1}x_{i}+b_{1}\right).$$

The encoding process from the hidden layer to the output layer:

(21) $$\hat{x}=g_{\theta_{2}}\left(h\right)=\sigma \left(W_{2}h+b_{2}\right).$$

RSSI data from multiple steps are embedded as input, represented as a two-dimensional matrix *x*, with dimensions indicating the number of time steps *t* and networks *n*. $x_{i}$ can be represented as:

(22) $$x_{i}=\left(\begin{array}{cccc} r_{i,1,1} & r_{i,1,2} & \cdots & r_{i,1,n}\\ r_{i,2,1} & r_{i,2,2} & \cdots & r_{i,2,n}\\ \vdots & \vdots & \ddots & \vdots \\ r_{i,t,1} & r_{i,t,2} & \cdots & r_{i,t,n} \end{array}\right)$$

where $r_{i,t,n}$ is the RSSI from the nth network in sample datum *i* at time step *t*.

Unlike traditional autoencoders, the improved method employs temporal distribution techniques tailored to the temporal characteristics of RSSI data, encoding and decoding data from each time step separately. To preserve the original input information, the output uses matrices of the same size and settings as the input. The hidden layer state g is extracted as the reconstruction input for the GRU for subsequent positioning.

The output of the positioning model consists of coordinates represented as a one-dimensional matrix *y*. In the positioning model, the optimization problem is described to adapt the positioning model to the training data and evaluate the model’s generalization ability. Specifically, the standard loss function in this problem is the mean square error (MSE), and the size of the error in actual predictions is represented by the mean absolute error (MAE), calculated as follows:

(23) $$MSE=\frac{1}{m}\sum_{i=1}^{m}{\left({\hat{y}}_{i}-y_{i}\right)}^{2}$$

(24) $$MAE=\frac{1}{m}\overset{m}{\underset{i=1}{\sum }}\left|{\hat{y}}_{i}-y_{i}\right|$$

## 4. Experiment and Result Analysis

**Figure 8:**
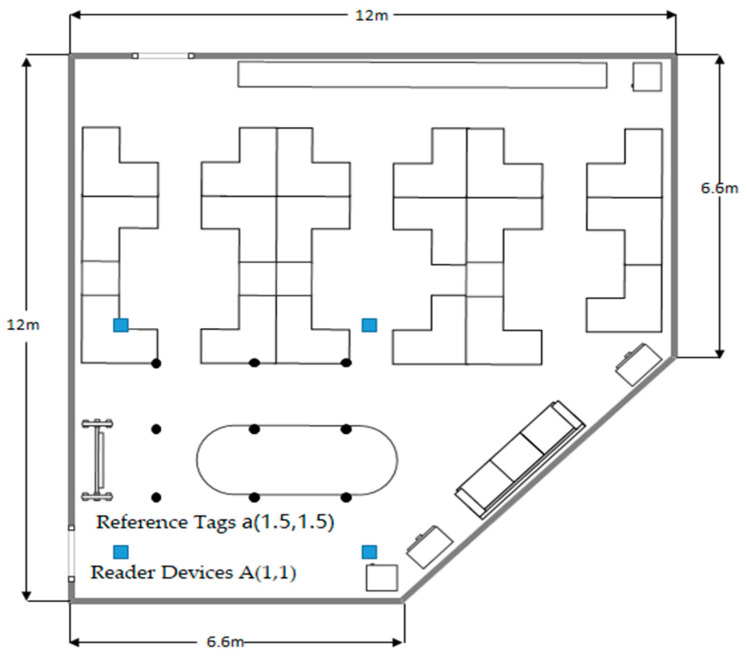
Experimental deployment.

In the deployment environment shown in Figure 8, data collection work is carried out. Readers are set up with reference tags, and reader devices are deployed at fixed positions spaced at intervals of 2 m and 5 m, as shown in the figure. In the preliminary phase, regular and automatic updates are made to collect the positions and RSSI values of the fixed reference tags as an offline fingerprint database. Then, dynamic scanning of the tags is performed as they move around an elliptical table from coordinates (3.5, 1.5) one full circle back to the initial position. The RSSI values received by the target tags at different positions during the movement process are continuously recorded. A total of 5000 sets of data samples is collected to determine their positional status.

When deploying the reference tags, several considerations and strategies were taken into account to improve the model’s localization performance:

1. Placement uniformity: Uniform placement of reference tags helps in creating a more reliable and accurate fingerprint database. The tags were placed at regular intervals to ensure consistent data collection across the deployment area;
2. Environmental characteristics: The indoor environment can introduce obstacles and sources of interference that affect RSSI readings. The tags were placed in various locations within the room to capture the impact of different environmental factors, such as furniture and walls, on the signal strength;
3. Height and orientation: The height at which tags are placed can affect the signal propagation. The tags were deployed at a consistent height, and their orientation was adjusted to optimize the signal reception;
4. Reducing multipath effects: Multipath effects can cause fluctuations in RSSI values. The deployment included the careful positioning of the tags to minimize the reflection and scattering of signals from surfaces;
5. Dynamic range of RSSI: It was ensured that the RSSI values cover a wide range to improve the robustness of the fingerprint database. The tags were placed at varying distances from the readers to collect a diverse set of RSSI readings.

By analyzing, studying, and filtering the real data collected from the RFID system, we determined the system and measurement noise levels. We improved the design by implementing an RSSI-based Kalman filter, which utilizes dynamic data information to obtain smooth numerical outputs, to eliminate the effects of noise. This approach enables noise estimation and the processing of tag information, and we verified the effectiveness and practicality of the improved algorithm.

**Figure 9:**
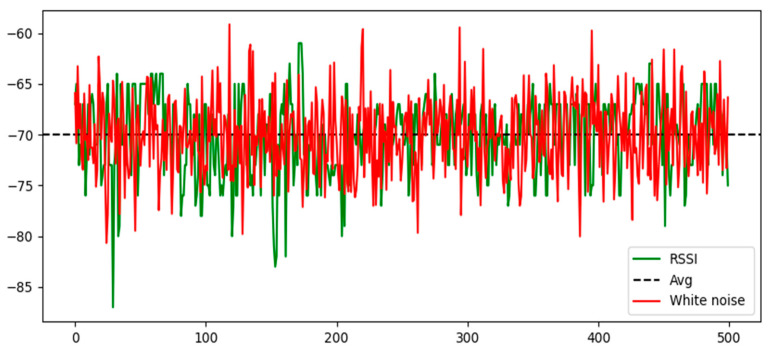
RSSI and white noise distribution.

We continuously recorded data sets of the target tags at the starting point during 500 readings. After receiving the measured values, we calculated the corresponding white noise distribution based on the read rate and observation noise, and then compared and plotted them as shown in Figure 9.

From Figure 9, it can be observed that the RSSI values exhibit characteristics such as white noise fluctuations, occasional abrupt value changes, and significant differences within certain intervals. Therefore, it is necessary to reduce the impurity noise in the collected data set through filtering algorithms.

**Figure 10:**
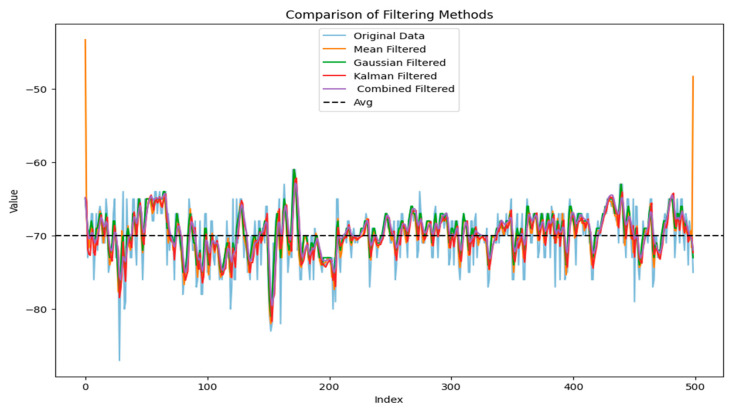
RSSI filtering effect chart.

**Figure 11:**
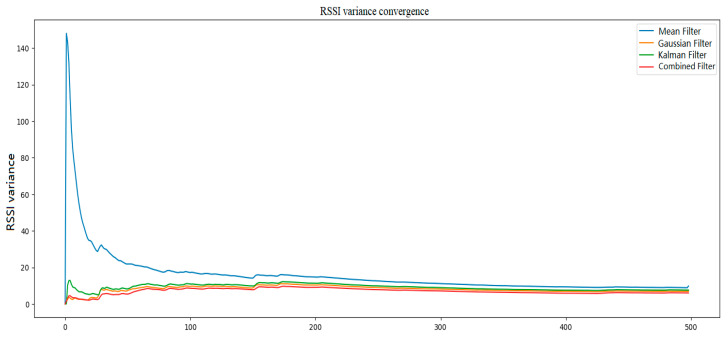
Filtering variance convergence.

Subsequently, after processing the data using Gaussian filtering, Kalman filtering, and a hybrid Kalman filtering algorithm, and calculating the variance convergence of the RSSI, Figure 10 and Figure 11 are obtained to evaluate the filtering effect.

**Figure 12:**
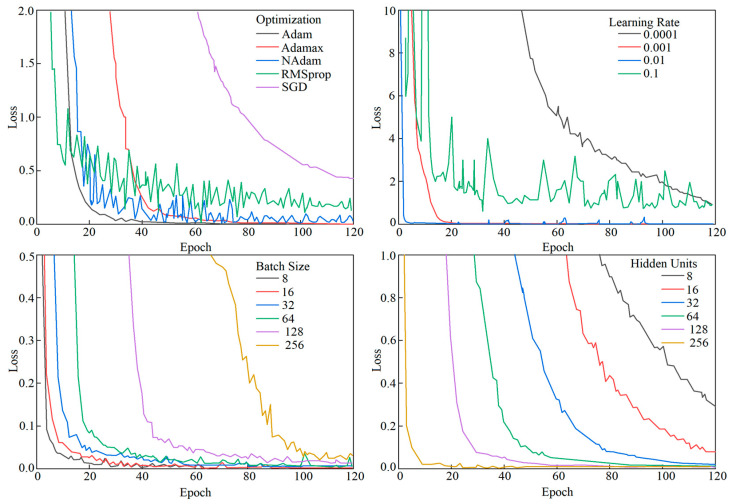
Loss function variation graph for each parameter.

From Figure 10 and Figure 11, it can be observed that Gaussian Kalman filtering effectively eliminates data outlier points and intervals, balances noise fluctuations in the signal, and provides a more accurate data output for subsequent positioning. Subsequently, by learning the features of the training set to obtain the model, in the training process, the model parameters can be set for convenience and the positioning-related performance can be evaluated. In this study, 80% of the collected dataset was used for training and 20% for testing. The mean square error loss function was selected as the model’s loss function. Different hyperparameters including the optimization algorithms, learning rate, batch size, and number of hidden layer neurons were adjusted. The variation in the loss values of the TAE-GRU model with increasing training epochs for each hyperparameter is shown in Figure 12.

The optimization algorithm adjusts the model parameters during training to minimize the loss function, thereby improving the model’s ability to fit the training data and enhance its performance. Observations show that the adaptive moment estimation (Adam) optimizer achieves faster convergence and exhibits fewer oscillations during training, making it more stable. The learning rate determines the step size of the parameter updates during training; a learning rate of 0.001 leads to rapid reduction in the loss function, ensuring model stability and faster convergence. The batch size determines the number of samples used for training in each parameter update. With a batch size of 64, the gradient of the loss function can be estimated more accurately, facilitating faster and more stable convergence to the optimal solution, which is critical for indoor positioning needs.

The number of neurons in the hidden layer determines the network’s learning capacity and complexity, directly influencing the model’s performance. A hidden layer neuron count of 128 shows an optimal performance, as the performance starts to saturate or decline beyond this point.

Following multiple trainings with the above hyperparameters, the trained model achieved a mean absolute error (MAE) of 0.293 m. To assess the accuracy and performance of the proposed method, we compared the TAE-GRU with the RNN, LSTM, and GRU using collected RSSI data for training and position estimation, summarizing and comparing the results of these methods throughout the process.

From Table 1, it can be observed that the TAE-GRU method used in this paper for RFID indoor localization shows significant advantages and performs well across various performance metrics. Specifically, from the perspective of localization accuracy, the TAE-GRU method exhibits improvements of 75.9%, 43.6%, 26.5%, and 9.2% compared to the NN, RNN, LSTM, and GRU methods, respectively. Compared to the RNN, LSTM, and GRU methods, the TAE-GRU method shows a notable performance improvement in localization time. Although the TAE-GRU localization method still has higher training and prediction times than the NN model, considering the requirements of accuracy and efficiency in localization, the TAE-GRU method still demonstrates good effectiveness and can be deployed effectively and rapidly in RFID localization systems.

**Figure 13:**
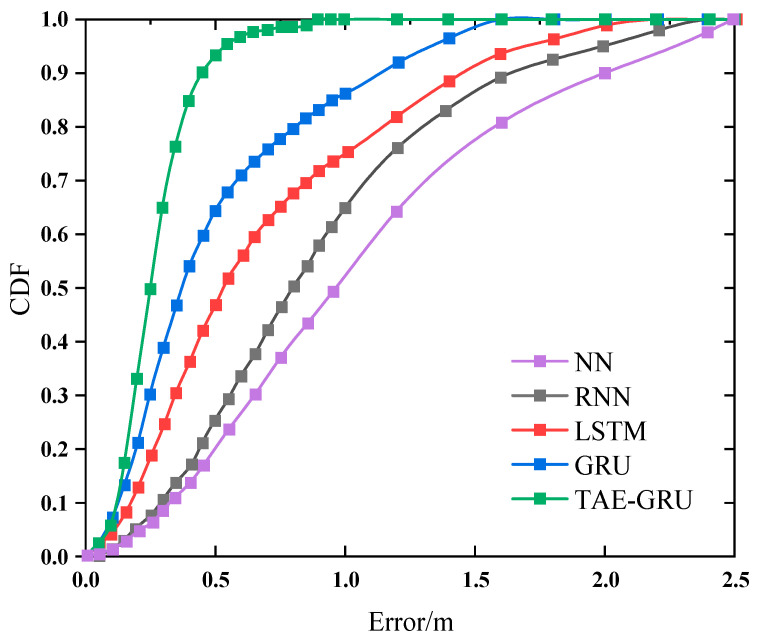
Cumulative distribution function of positioning method error.

Figure 13 shows the CDF of all positioning methods. From the comparison data, it can be seen that the error of the proposed TAE-GRU method is less than 1.0 m for 99% of the cases, and less than 0.5 m for 80% of the cases. The TAE-GRU recurrent neural network positioning method proposed in this chapter has improved on the positioning accuracy of the other methods to some extent. The trained algorithm can be applied more quickly in indoor scenarios.

## 5. Future Directions

This study mainly discusses the effectiveness of the TAE-GRU-based localization method and some limitations that need to be resolved to enhance its generalizability and scalability. These limitations include issues with the controlled environment, such as the space size, building and furniture layout, deployment locations for reference tags, etc.; an inability to generalize well to other areas or buildings due to differences in environmental factors, such as obstacles, signal reflections, interference sources, etc.; the need for repeated training processes, which limits its scalability and practical application. As well, the model’s performance in non-line-of-sight (NLOS) scenarios was not considered, which is common in real-world applications and can significantly impact the localization accuracy.

Future work will focus on addressing these limitations and expanding the applicability of our approach. This includes testing and validating the proposed method in diverse environments, extending the experimental setup to various indoor environments, including different types of buildings and layouts, and exploring transfer learning techniques to adapt the model to new environments with minimal retraining. In addition, we will investigate methods to reduce the need for extensive retraining, including developing adaptive algorithms that can fine-tune the model with a smaller dataset from the new environment and exploring incremental learning techniques that allow the model to update its parameters dynamically as new data becomes available. We will also test the algorithm in NLOS conditions to evaluate its performance and identify any necessary adjustments, as well as develop strategies to mitigate the effects of NLOS on RSSI measurements.

## 6. Conclusions

This study demonstrates that the TAE-GRU model substantially advances the accuracy and efficiency of RFID-based indoor localization systems. By incorporating Gaussian Kalman filtering to mitigate noise and employing the strengths of GRU networks in handling sequential data, the proposed method outperforms traditional techniques. The experimental results reveal that our approach surpasses conventional neural networks, the RNN, LSTM, and standard GRU models, in terms of both localization accuracy and processing speed. These findings affirm the efficacy and practical applicability of the TAE-GRU model in indoor localization contexts.

Future research endeavors will concentrate on further refining the TAE-GRU model to adapt to more complex indoor environments and varying operational conditions. Exploring the incorporation of additional sensory data and advanced machine learning techniques holds potential for further enhancements in the model’s localization accuracy and robustness. Additionally, extending the application of this approach to broader domains, such as smart buildings and internet of things (IoT) systems, will be a focal point of future investigations.

## Figures and Tables

**Table 1:** Comparison of Localization Methods.

Model	Training Time (s)	Testing Time (s)	MAE(m)	Improvement in Accuracy (%)
NN	24	0.010	1.214	0
RNN	49	0.043	0.822	32.3%
LSTM	214	0.146	0.614	49.4%
GRU	68	0.076	0.404	66.7%
TAE-GRU	30	0.023	0.293	75.9%

## Data Availability

The data used to support the findings of this study are available from the corresponding author upon request.
